# Surgical treatment in hepatic trauma: factors associated with hospitalization time

**DOI:** 10.1590/0100-6991e-20202874

**Published:** 2021-03-17

**Authors:** PAULA DE OLIVEIRA TRINTINALHA, EMANUELLA ROBERNA INÁ CIRINO, RENATA FERNANDA RAMOS MARCANTE, GABRIEL RAMOS JABUR, PATRÍCIA LONGHI BUSO

**Affiliations:** 1 - Hospital Universitário Cajuru, Cirurgia Geral - Curitiba - PR - Brasil; 2 - Universidade Estadual do Oeste do Paraná, Curso de Medicina - Francisco Beltrão - PR - Brasil

**Keywords:** Liver, Wound and Injuries, Abdominal Injuries, Hospitalization Time, Fígado, Ferimento e Lesões, Traumatismos Abdominais, Tempo de Internação

## Abstract

**Objective:**

the aim of this study was to identify associated factors with the increased length of hospital stay for patients undergoing surgical treatment for liver trauma, and predictors of mortality as well as the epidemiology of this trauma.

**Methods:**

retrospective study of 191 patients admitted to the Cajuru University Hospital, a reference in the treatment of multiple trauma patients, between 2010 and 2017, with epidemiological, clinicopathological and therapeutic variables analyzed using the STATA version 15.0 program.

**Results:**

most of the included patients were men with a mean age of 29 years. Firearm injury represents the most common trauma mechanism. The right hepatic lobe was injured in 51.2% of the cases, and hepatorraphy was the most commonly used surgical correction. The length of hospital stay was an average of 11 (0-78) days and the length of stay in the intensive care unit was 5 (0-52) days. Predictors for longer hospital stay were the mechanisms of trauma, hemodynamic instability at admission, number of associated injuries, degree of liver damage and affected lobe, used surgical technique, presence of complications, need for reoperation and other surgical procedures. Mortality rate was 22.7%.

**Conclusions:**

the study corroborated the epidemiology reported by the literature. Greater severity of liver trauma and associated injuries characterize patients undergoing surgical treatment, who have increased hospital stay due to the penetrating trauma, hemodynamic instability, hepatic packaging, complications and reoperations.

## RESUMO

**Objetivo:**

identificar fatores associados ao aumento do tempo de hospitalização de pacientes submetidos a tratamento cirúrgico por trauma hepático e descrever preditores de mortalidade, assim como a epidemiologia desse trauma.

**Métodos:**

estudo retrospectivo de 191 pacientes admitidos no Hospital Universitário Cajuru, referência no atendimento de politraumatizados, no período entre 2010 e 2017, com variáveis epidemiológicas, clinicopatológicas, terapêuticas analisadas por meio do programa STATA versão 15.0.

**Resultados:**

maioria dos pacientes incluídos eram homens com média de idade de 29 anos. Ferimento por arma de fogo representou o mecanismo de trauma mais comum. O lobo hepático direito foi lesado em 51,2% dos casos e hepatorrafia foi a correção cirúrgica mais empregada. O tempo de internamento hospitalar foi em média de 11(0-78) dias e o tempo de internação em unidade de terapia intensiva de 5 (0-52) dias. Preditores de maior tempo de hospitalização foram mecanismo de trauma, instabilidade hemodinâmica à admissão, número de lesões associadas, grau da lesão hepática e lobo acometido, técnica cirúrgica empregada, presença de complicações, necessidade de reoperação e outros procedimentos cirúrgicos. Taxa de mortalidade foi de 22,7%.

**Conclusões:**

o estudo corroborou a epidemiologia descrita na literatura. Maior gravidade do trauma hepático e das lesões associadas caracterizam os pacientes submetidos ao tratamento cirúrgico, que apresentam aumento de tempo de hospitalização devido a trauma penetrante, instabilidade hemodinâmica, tamponamento hepático, complicações e reoperações.

## INTRODUCTION

Hepatic trauma corresponds to 5% of admissions in reference centers for the care of the polytraumatized^1^ ^-^ ^3^. Abdominal traumas especially affect the liver due to its size and anatomical position, the most common mechanism being penetrating trauma^3^ ^-^ ^6^.

Treatment of hepatic trauma may be non-operative or surgical, according to hemodynamic status, associated lesions, and degree of injury, according to the classification of the American Association for the Surgery of Trauma (AAST)^6^. In the last years, non-operative treatment has gained space in the management of hepatic trauma due to greater accessibility to imaging^7^ ^,^ ^8^. The non-operative treatment of any solid viscus needs to be performed in a center with availability of imaging, intensive care unit (ICU), and surgical staff^9^.

Surgical treatment is usually indicated for patients who are hemodynamically unstable, with signs of peritonitis or injury to other intra-abdominal structures, or when non-operative management has failed. The objective is to control bleeding and to repair lesions, favoring the survival of critically ill patients, even if associated with a greater number of complications, such as hepatic abscess and biliary fistulas^4^ ^,^ ^8^ ^,^ ^10^.

Considering the high morbidity and mortality and the high costs for public health when caring for the polytraumatized, we sought to identify predictors of increased hospitalization time of hepatic trauma patients undergoing surgical treatment, as well as to describe its epidemiology and to identify factors associated with higher mortality. 

## METHODS

We conducted a retrospective study by reviewing records of patients with hepatic trauma who underwent surgical treatment from 2010 to 2017 at the General Surgery Service of the Cajuru University Hospital, in Curitiba, state of Paraná, Brazil. This study was approved by the Ethics in Research Committee of the Pontifical Catholic University of Paraná (CAAE no 08547119.2.0000.0020). We included all patients undergoing surgical treatment for hepatic trauma in this period, excluding those whose records were incomplete.

The variables analyzed were sex, age, mechanism of trauma, hemodynamic stability, or its lack thereof, imaging tests, and laboratory tests (hemoglobin, lactate, and base deficit) at admission, as well as injury severity, according to the classification of the American Association for the Surgery of Trauma, and the affected segments. We used the anatomical divisions and nomenclatures proposed by Coinaud^11^. By this segmentation scheme, the left hepatic lobe contains segments II, III, and IV and the right, segments V, VI, VII and VIII. Hepatic segment I - caudate lobe - is considered apart from the lobe’s division.

We also analyzed the presence of associated lesions, surgical technique employed in the injury management, the need for other surgical procedures, reoperation, as well as the length of ICU and total hospital stay, and death. We considered hemodynamic instability a systolic blood pressure (SBP) less than 90 mmHg at admission.

We divided the patients into two groups: the first consisting of patients who were discharged from the hospital and the second with those who died. The division was performed to reduce the confounding bias for analysis of factors associated with longer hospitalization time.

We described results as means, medians, minimum and maximum values, and standard deviations (quantitative variables), or by frequency and percentages (categorical variables). To evaluate the factors (variables of interest), we used the chi-square test for categorical variables and the nonparametric Kruskal Wallis and Mann-Whitney tests (with significance level corrected by Bonferroni) to analyze the difference of quantitative variables between groups. We chose the nonparametric approach because the data did not display normality by the Kolmogorov-Smirnov test. Values of p<0.05 indicated statistical significance. We analyzed the data with the Stata software, version 15.0.

## RESULTS

We included 191 records in the study, of whom we excluded two due to incomplete data. Of the 189 remaining cases, the majority were male (90.5%), and the mean age was 29 years (11-75). The most common mechanism of trauma was gunshot wound (59.8%), followed by stabbing wound (25.4%), automobile accidents (11.1%), trampling (2.1%), and fall from height (1.6%) (Table 1). 

**Table 1:** Characteristics of the patients undergoing hepatic lesion surgical treatment according to the outcomes.

Total sample	Outcome hospital discharge (n = 149)	Outcome death (n = 40)	p-Value
Characteristics			
Age			
Median	28.68	33.83	0.207
Reach	11-63	16-75	
Gender			
Female	15 (10%)	3 (7.5%)	
Male	134 (89.9%)	37 (92.5%)	0.769
Mechanism of trauma			
Penetrating trauma	133 (89.3%)	28 (70%)	<0.001
Stabbings	47 (31.5%)	1 (2.5%)	
Gunshot wounds	86 (57.7%)	27 (67.5%)	
Blunt trauma	16 (10.7%)	12 (30%)	
Automobile acidents	12 (8.1%)	9 (22.5%)	
Trampling	4 (2.7%)	0	
Fall from height	0	3 (7.5%)	
Degree of liver injury			
Grade I	31 (22.5%)	3 (8.8%)	0.007
Grade II	43 (31.2%)	4 (11.8%)	
Grade III	40 (29%)	14 (41.2%)	
Grade IV	20 (14.5%)	12 (35.3%)	
Grade V	4 (2.9%)	1 (2.9%)	
Hepatic lobe			
Right	54 (52.9%)	12 (44.4%)	0.592
Left	33 (32.4%)	9 (33.3%)	
Both	15 (14.7%)	6 (22.2%)	

Eighty-five patients (45%) had hemodynamic instability at admission. The time of hospitalization in the intensive care unit (ICU) varied between zero and 52 days, with mean of five days, and the hospital stay, between zero and 78 days, with mean of 11 days. Death was the outcome of 43 patients (22.7%).

The right hepatic lobe was affected in 51.2% of cases, the left in 32.6%, and both in 16.3%. There was evaluation of lesion severity in 172 individuals, the most common being grade III (31.4%) (Table 1). Associated lesions in other organs occurred in165 patients (87.3%) (Table 2). 

**Table 2:** Lesions associated with the hepatic trauma.

Topography	Total sample	Outcome hospital discharge (n =128)	Outcome death n = 37	p-Value
Cervical				
Cervical vessels		4 (2.7%)	2 (5%)	0.703
Chest				
Chest wall		31 (20.8%)	11 (27.5%)	0.394
Heart		4 (2.7%)	2 (5%)	0.608
Lung		19 (12.8%)	9 (22.5%)	0.136
Esophagus		0	3 (7.5%)	0.009
Diafragm		60 (40.3%)	12 (30%)	0.274
Thoracic vessels		2 (1.3%)	2 (5%)	0.197
Abdomen				
Bile duct		7 (4.7%)	6 (15%)	0.033
Spleen		19 (12.8%)	5 (12.5%)	1
Stomach		29 (19.5%)	6 (15%)	0.649
Duodenum		17 (11.4%)	8 (20%)	0.148
Small intestine		21 (14.1%)	15 (37.5%)	0.002
Pancreas		13 (8.7%)	3 (7.5%)	1
Colon		25 (16.8%)	10 (25%)	0.255
Adrenal		0	1 (2.5%)	0.212
Kidneys		27 (18.1%)	8 (20%)	0.820
Ureters		0	1 (2.5%)	0.212
Rectum		1 (0.7%)	0	1
Bladder		2 (1.3%)	1 (2.5%)	0.512
Abdominal vessels		17 (11.4%)	7 (17.5%)	0.295

Hepatorrhaphy was the only surgery performed in most patients (91.5%), as shown in table 3. Damage control surgery was required in 21.7% of patients and thoracotomy with clamping of the thoracic aorta, in 5.3%. During the same surgical act, 59 individuals required other surgical procedures to treat other lesions.

**Table 3:** Length of hospital and ICU stay of those patients who were discharged from the hospital.

Variable		ICU length of stay			Hospital length of stay		
		n	Average ± SD Median (min-max)	p-Value	n	Average ± SD Median (min-max)	p-Value
Types of lesions	Contusion	16	14.19 ± 13.49 13.5 (0-52)	<0.001*	16	29 ± 23.29 24 (1-78)	0.001*
	Perforant	133	3.5 ± 6.63 0 (0-45)		133	10.44 ± 8.30 8 (2-60)	
Hemodynamic instability at admission		50	6.92 ± 7.31 4.5 (0-26)	<0.001*	50	16.1 ± 13.76 10 (1-78)	<0.001*
Affected hepatic lobe	Right	54	45.96 ± 8.38 3 (0-39)	0.002**	54	13.76 ± 11.81 9 (1-54)	0.002**
	Left	33	0.88 ± 1.6 0 (0-6)		33	6.30 ± 3.07 6 (2-13)	
	Both	15	4 ± 5.03 3 (0-18)		15	15.53 ± 18.13 9 (5-78)	
Surgical technique	Hepatorraphy	141	4.26 ± 7.35 0 (0-45)	0.216*	141	11.95 ± 11.29 8 (1-78)	0.376*
	Intrahepatic balloon	4	15.25 ± 17.37 11 (0-39)	0.092*	4	21.75 ± 13.05 19.5 (9-39)	0.045*
	Hepatectomy	4	19.5 ± 22.58 13 (0-52)	0.060*	4	28.00 ± 28.46 17.5 (7-70)	0.086*
	Cauterization	10	7 ± 8.11 4 (0-24)	0.127*	10	12.27 ± 11.8 11 (3-39	0.211*
	Damage control	21	14.38 ± 11.63 1 (2-45)	<0.001*	21	22.90 ± 14.38 17 (5-60)	<0.001*
Occurrence of complications	Yes	44	9.86 ± 10.75 6 (0-52)	<0.001*	44	21.55 ± 16.02 16 (7-78)	<0.001*
Reoperation	Yes	32	12.78 ± 12.68 10.5 (0-52)	<0.001*	32	24.38 ± 17.84 19 (4-78)	<0.001*

Complications occurred in 40.2% of patients, the non-surgical ones being the most frequent (28.6%). Among the surgical, sepsis with abdominal focus was the main one (23 patients, 12.2%), followed by biliary fistula (9.5%) and intra-abdominal abscess (4.8%). A new operative intervention was performed in 22 patients due to complications, excluding damage control reoperations.

Longer hospital stay was associated with the mechanism of trauma, hemodynamic instability at admission, technique used, need for reoperation, number of associated lesions, need for other surgical procedures, degree of liver injury, and affected liver lobe. Regarding the blood markers, there was a positive correlation between the time of hospitalization with the values of lactate and base deficit, and a negative correlation with hemoglobin values (Table 3). There was no significant difference regarding sex and imaging at admission.

As for the affected liver lobe, we observed that among the patients who were discharged, the presence of a sole lesion in the left lobe was a factor for shorter stay, both the hospital one and in the ICU, when compared to those who had only the right lobe affected and those injured in both lobes (Table 3).

Patients undergoing hepatorrhaphy and intra-hepatic balloon insertion also displayed longer hospital stay than those not submitted to such technique, though with no difference for the ICU stay time. It is worth noting that this technique was not used in isolation in any patient.

When considering each variable individually, we found that the factors significantly associated with death were hemodynamic instability at admission (p<0.01), lower hemoglobin (p<0.001), higher base deficit (p=0.001), higher lactate (p=0.005), need for damage control surgery (p<0.05), and the trauma mechanisms gunshot wounds, automotive accidents, and falls (p<0.001). In addition, higher number of associated lesions (p=0.008), especially if located in the esophagus, bile duct, or small intestine (p=0.009, p=0.033, and p=0.002, respectively) were also risk factors. Presence of complications (p<0.001), non-surgical complications (p=0.001), and sepsis of abdominal focus (p<0.001) were also associated with the outcome death. Grades III and IV hepatic lesions were more present among those who died, though not significant (p=0.07). 

When comparing the outcome with the type of operation performed, we observed a significant association between deaths and liver packing (45, p<0.001) (Table 1). We found the same association for individuals submitted to hepatic cauterization (25%, p=0.002), thoracotomy with aortic clamping (25%, p<0.001), cavity drainage (60%, p=0.031), and damage control surgery (50%, p<0.001). We highlight that patients who died underwent a significantly higher number of surgical procedures.

## DISCUSSION

Liver trauma is more common in men aged 20 to 40 years^4^ ^,^ ^12^. Blunt mechanisms are more frequent than penetrating ones^4^ ^,^ ^12^. However, in patients undergoing surgical treatment, the penetrating mechanism is the most common (78.5%)^10^. The right lobe of the liver is the most affected^9^ ^,^ ^10^. Our findings corroborate these data.

Tarchouli et al. carried out a retrospective analysis and found a higher incidence of grade I liver lesions (81.9%), followed by grades II (29.6%) and III (24.3%)^4^. Bernardo et al found higher rates of grade III liver lesions (39.2%). Probably, this difference occurred because the former study analyzed liver trauma in general, while the latter studied only patients with operated liver injuries^12^. Like the latter, most patients in our series had grade III liver injuries. More severe liver lesions are more frequent in those undergoing surgical treatment.

In several publications, most patients 67.5% - 86% undergoing surgical treatment had associated lesions^4^ ^,^ ^10^ ^,^ ^13^. The most affected extra-abdominal organs are the thoracic organs, and the most affected abdominal viscera are the spleen and kidney^4^ ^,^ ^13^. Differently from the literature, in our study, the most common intra-abdominal associated injured organs were the stomach and small intestine. Worse prognoses were associated with injuries to the esophagus, biliary tree, and small intestine.

The indication of surgical treatment of the patients in this study was related to hemodynamic instability at admission or to the need to approach other associated lesions, as seen in other series^4^ ^,^ ^10^. The most used surgical techniques are hepatorrhaphy (38.5% - 80%), damage control (6.54% - 26.9%), electrocauterization (28% - 8.9%), intrahepatic balloon (6.8%), and hepatectomy (0.9% - 3.8%)^1^ ^,^ ^4^ ^,^ ^10^ ^,^ ^13^. In our study, hepatorrhaphy was also the most used surgical technique. However, we observed that patients with worse outcome underwent a greater variety of techniques, probably due to greater severity. The absence of trauma severity indices represents a confounding bias since we could not study the injuries according to severity.

The most frequent post-surgical complications are surgical wound dehiscence (48.8%), non-surgical complications (29.8%) - pneumonia and urinary tract infection -, and bilomas and/or pancreatic fistulas (14.8%)^14^. The mortality rate of liver trauma ranges from 4.5% to 40%, correlating with severe injuries (grades III, IV, V, and VI), hemorrhagic shock, multiple associated lesions, and the mechanism of trauma, being higher when treatment is surgical^1^ ^,^ ^10^ ^,^ ^13^ ^,^ ^14^.

The duration of hospitalization of individuals with liver trauma can vary between two and 42 days, with an average of 10 days of hospitalization in the intensive care unit^14^. Among patients treated with surgical intervention, the duration of hospitalization varies between one and 39 days (mean 12)^4^. The mean hospitalization and ICU times in the present study were similar.

Factors associated with mortality are gunshot wounds, hemodynamic instability at admission, grades IV and V lesions, associated lesions^9^, need for massive blood transfusion, perioperative complications, and low hemoglobin values at admission^6^. Some variables are associated with a higher risk of liver complications after surgical treatment, such as age over 60 years and more complex surgical procedures (damage control, Pringle maneuver, intrahepatic balloon, and vascular access to the hepatic veins)^2^. In the literature review performed, we found no factors directly related to hospitalization time.

We observed that the increase in hospitalization time is directly proportional to the increase in injury severity, the greater number of associated lesions, the increase in the number of liver surgical techniques, and the number of associated non-hepatic procedures. We also identified shorter hospital stay in those patients with an isolated lesion of the left hepatic lobe. Patients with hemodynamic instability at admission, lower hemoglobin, higher base deficit, need for damage control surgery, and with more severe trauma mechanisms, such as gunshot wounds, are more likely to die. Non-surgical complications and sepsis with abdominal focus were associated with late death, being related to longer hospital stay.

The lack of trauma severity indices and sample size are the main limitations of the present study. There is a need for further studies to better elucidate the factors associated with longer hospitalization time of patients undergoing surgical treatment of liver injuries.

## CONCLUSION

Patients undergoing surgical treatment of liver injury are essentially those with greater trauma severity and associated injuries. Longer hospitalization time is associated with complications, penetrating trauma, lesions of the right hepatic lobe, and associated lesions. The number of surgical techniques employed was a predictor of mortality. Lactate and base deficit are directly proportional to the time of hospitalization, while hemoglobin values are inversely proportional.
